# Vitamin A deficiency during pregnancy of HIV infected and non-infected women in tropical settings of Northwest Ethiopia

**DOI:** 10.1186/1471-2458-11-569

**Published:** 2011-07-15

**Authors:** Andargachew Mulu, Afework Kassu, Kahsay Huruy, Birhanemeskel Tegene, Gashaw Yitayaw, Masayo Nakamori, Nguyen Van Nhien, Assegedech Bekele, Yared Wondimhun, Shigeru Yamamoto, Fusao Ota

**Affiliations:** 1 Department of Microbiology, Immunology and Parasitology, College of Medicine and Health Sciences, University of Gondar, P. O. Box 196, Gondar, Ethiopia; 2 Institute of Virology, Faculty of Medicine, University of Leipzig, Johannisallee 30, 04103, Leipzig, Germany; 3 Department of Medical Laboratory Technology, College of Medicine and Health Sciences, University of Gondar P. O. Box 196, Gondar, Ethiopia; 4 Division of Nutrition and Food Science, Ochanomizu University, Tokyo 112-8610, Japan; 5 Department of Science and Network Direction, National Institute for Food Control, 15A Phan Huy Chu, Hanoi, Vietnam; 6 Department of Human Anatomy, College of Medicine and Health Sciences, University of Gondar, P.O. Box 196, Gondar, Ethiopia; 7 Department of Medicine, Howard University Hospital, Howard University; 8 International Nutrition, Department of Food and Nutritional Sciences, Graduate School of Human Life Sciences, Jumonji University, 2-1-28 Sugasawa, Niiza-City, Saitama 352-8510, Japan; 9 Department of Preventive Environment and Nutrition, Institute of Health Biosciences, The University of Tokushima, Japan

**Keywords:** Vitamin A deficiencies, pregnancy, HIV infection, Ethiopia

## Abstract

**Background:**

Vitamin A deficiency (VAD) is known to be a major public health problem among women of reproductive age in South East Asia and Africa. In Ethiopia, there are no studies conducted on serum vitamin A status of HIV-infected pregnant women. Therefore, the present study was aimed at determining the level of serum vitamin A and VAD among pregnant women with and without HIV infection in tropical settings of Northwest Ethiopia.

**Methods:**

In this cross-sectional study, blood samples were collected from 423 pregnant women and from 55 healthy volunteers who visited the University of Gondar Hospital. Serum concentration of vitamin A was measured by high performance liquid chromatography.

**Results:**

After controlling for total serum protein, albumin and demographic variables, the mean ± SD serum vitamin A in HIV seropositive pregnant women (0.96 ± 0.42 μmol/L) was significantly lower than that in pregnant women without HIV infection (1.10 ± 0.45 μmol/L, P < 0.05). Likewise, the level of serum vitamin A in HIV seropositive non-pregnant women (0.74 ± 0.39) was significantly lower than that in HIV negative non-pregnant women (1.18 ± 0.59 μmol/L, P < 0.004). VAD (serum retinol < 0.7 μmol/L) was observed in 18.4% and 17.7% of HIV infected and uninfected pregnant women, respectively. Forty six percent of non-pregnant women with HIV infection had VAD while only 28% controls were deficient for vitamin A (P = 0.002).

**Conclusion:**

The present study shows that VAD is a major public health problem among pregnant women in the tropical settings of Northwest Ethiopia. Considering the possible implications of VAD during pregnancy, we recommend multivitamin (which has a lower level of vitamin A) supplementation in the care and management of pregnant women with or without HIV infection.

## Background

Vitamin A deficiency (VAD) is known to be a significant public health problem around the world and it is particularly serious among women of reproductive age in South-East Asia and Africa [1-4]. It has now become evident that VAD in women has negative consequences on their health status as well as on their infants [3,4]. The link between VAD morbidity and mortality from infectious diseases [5] and non-infectious diseases [6-8] has been known for several years.

VAD in pregnant women is associated with night blindness, severe anaemia, wasting, malnutrition, and reproductive and infectious morbidity [9], and increased risk of mortality 1-2 years following delivery [4]. VAD reduces lymphocyte response [10], and also leads to reduced levels of secretory IgA in mucous membranes and therefore weakens the local barriers to infection [7,11,12]. As a result, vitamin A deficient women were found to be more susceptible to illnesses of both infectious such as frequent infection of mucous surface of hallow viscera [4] and non-infectious (eclampsia, pre-eclampsia, premature rupture of membrane) diseases [6,13,14].

In the era of HIV/AIDS, vitamin A was also postulated to reduce mother to child transmission (MTCT) of HIV by affecting several maternal, fetal, and/or child risk factors for transmission, including the clinical, immunological, or viral stage of HIV disease among pregnant women. Vitamin A has an effect on the integrity of the epithelial lining of the placenta, maternal lower genital tract, or breast. Its deficiency leads to the occurrence of prematurity and low birth weight, and the status of the systemic and digestive mucosal immune systems of the fetus and the child [15,16]. Observational studies in sub-Saharan Africa have shown that, low serum vitamin A levels in HIV-infected women to be associated with significantly increased rates of MTCT of HIV [17,18] and infant mortality [17,19]. On the contrary, randomized trials of vitamin A supplementation have found that, vitamin A supplementation increases the risk of MTCT [20,21] and can increase mortality in some children born to HIV positive mothers. Vitamin A supplementation in HIV-infected children, on the other hand, has been associated with protective effects against mortality and morbidity, similar to that seen in HIV-negative children [22].

In Ethiopia, studies on VAD in the general population and among pregnant women are scant, despite decades of documentation of VAD as a major public health problem affecting up to 40% of pregnant women [23-25]. Recently, VAD has also been reported as a severe public health problem among tuberculosis [26] and diarrheic [27] patients infected with HIV. There are no studies examining serum vitamin A status of HIV-infected pregnant women in northwest Ethiopia. Therefore, the present study aimed to determine the level of serum vitamin A among pregnant women with and without HIV infection in tropical settings of Northwest Ethiopia.

## Methods

### Subjects and settings

Pregnant women who visited the antenatal clinic of the University of Gondar Hospital between March and June 2005 in their first trimester for routine antenatal care follow-ups were approached for recruitment. The Hospital is a tertiary level teaching and service rendering hospital that provides health service for over 5 million inhabitants in Northwest Ethiopia. Healthy non pregnant women, who were living in the same geographic locale as pregnant participants, were recruited from voluntary blood donors and served as control subjects. Informed consent was obtained from the study participants and approved by the Research Ethics Committee of the University of Gondar. None of the subjects had cirrhosis and none of them received vitamin A supplementation.

### Anthropometric data

Body weight and height was measured to the nearest 0.1 kg on an electronic digital scale and to the nearest 0.1 cm, respectively. Body mass index (BMI) was calculated and used to determine the nutritional status of the study subjects [28], though it is not a surrogate for nutritional assessment during pregnancy.

### Blood collection and HIV screening

About 5 ml of venous blood was collected from each subject as per the routine antenatal care follow up of the pregnant women in the morning but fasting status was not ascertained. After clot was retracted, the blood samples were centrifuged and sera were separated from the cells following standard procedures and stored at -40°C until tested. The sera were tested for presence of HIV-1 antibodies using rapid HIV-1 diagnostic test kits (Abbott, Belgium) with a sensitivity of 99.9% and specificity of 98% following the manufacturers' instructions. The results were interpreted following the current national algorithm for screening of HIV-1 infection [29]. CD4 cells count was not measured and none of the study subjects was on antiretroviral therapy because of lack of access in the setting during the study period. Pre and post HIV test counselling was provided to patients and controls as per the routine program.

### Biochemical analysis

Serum level of retinol (vitamin A), albumin, total protein and total cholesterol were determined following standard procedures and briefed as follows:

*Serum vitamin A: *Serum retinol was determined using high performance liquid chromatography (HPLC) according to the method of Arroyave *et al *[30] utilizing a Shiseido HPLC system (Shiseido Co. Ltd., Tokyo, Japan) which consisted of a separation by a reverse phase column (Capcell Pak C18 MG S-5, 3 × 250 mm, 5 μm, Shiseido, Japan) and ultraviolet detection using methanol as a mobile phase system. The flow rate was adjusted at 500 μl/min with column temperature set at 40°C. Serum was deproteinised with an alcoholic solution of retinyl acetate and extracted into hexane. The organic layer was separated, evaporated to dryness under a steam of nitrogen, reconstituted in methanol and injected into the HPLC system with a detection wave length set at 325 nm. All extraction procedures were carried out under reduced light in order to prevent oxidation of the compounds. Pooled human sera were used to measure intra and inter-assay coefficients of variation in laboratory analyses for serum retinol and found to be 6.5% and 3.3%, respectively. VAD was defined as serum retinol level below <0.7 μmol/l or 20 mg/dl [1].

### Serum albumin

Serum albumin was determined by end point colorimetric assay in which at pH value of 4.2 albumin display a sufficiently cationic character to be able to bind with bromcresol green (BCG), an anionic dyestuff, to form a blue green complex. The intensity of the blue green colour is directly proportional to the concentration of albumin and was determined photometrically (AUTOLAB PM 4000/3, Analyser Medical System, Italy). The analytical sensitivity (lower detection limit) of the assay which represents the lowest measurable albumin concentration that can be distinguished from zero is 0.2 g/dl or 2 gm/l. Hypoalbuminemia was defined as serum albumin level below 3.5 gm/dl or 35 gm/l [31].

### Total Protein

Total serum protein (TSP) was determined by enzymatic reaction sequence in which protein in serum forms a blue coloured complex when reacted with cupric ions in an alkaline solution (Teco Diagnostics, USA). The intensity of the violet colour was proportional to the amount of protein present when compared to a solution with known protein concentration and was determined photometrically (AUTOLAB PM 4000/3, Analyser Medical System, Italy). Hypoproteinemia was defined as serum protein level below 6.2 gm/dl or 62 gm/l [31].

### Total Cholesterol

Total cholesterol was determined by enzymatic colorimetric test using cholesterol esterase and cholesterol oxidase. Briefly, cholesterol was converted to cholest-4-en 3-one and hydrogen peroxide by oxygen with the aid of cholesterol esterase. The produced hydrogen peroxide under the catalytic action of peroxidise formed a red dyestuff when reacted with 4- aminoantipyrine and phenol (Biocon Diagnostic, Germany). The colour intensity was directly proportional to the concentration of cholesterol and was determined photometrically (AUTOLAB PM 4000/3, Analyser Medical System, Italy). The analytical sensitivity (lower detection limit) of the assay which represents the lowest measurable cholesterol concentration that can be distinguished from zero was mg/dl (0.08 mmol/Lt). Hypocholesterolemia was defined as serum level below 154 mg/dl [31]. Cholesterol level of below 200 mg/dl was considered as indicator for the absence of lipid metabolic disturbance and cholesterol level of 200-300 mg/dl with HDL cholesterol below 35 mg/dl and above 300 mg/dl is considered as indicator for the presence of lipid metabolic disturbance.

### Quality control

In all cases commercially available control materials with established values were used for quality control purpose and no failure was observed to obtain the assigned values. In addition, pooled sera were used to measure intra and inter-assay coefficients of variation in laboratory analyses for serum retinol and found to be less than 5%. Sufficient sera for the determination of albumin, protein and cholesterol were only found for the 107 pregnant women. And also it was not possible to find extra sera in the control group.

### Data analysis

Data were analyzed using SPSS version 17 statistical package (SPSS, Inc., Chicago, IL, USA). The significance of differences in the serum vitamin A, total protein, albumin and total cholesterol levels between pregnant women with or without HIV infection and healthy controls was evaluated using a one way analysis of variance with post hoc Tukey test to determine pairs of means which differ significantly. In all cases a P value of < 0.05 was considered statistically significant.

## Results

A total of 423 pregnant women were included in this study. Among the 423 pregnant women, 44 (10.4%) were infected with HIV. From the total of 55 non pregnant voluntaries blood donors, who were living in the same geographic locale with the pregnant women, 30 (54.5%) were found to be HIV positive. The mean age of pregnant and the non pregnant women was 25.38 ± 5.68 (range 16-45 years) and 28.07 ± 7.2 (range 18-41 years), respectively. The mean age of pregnant women with and without HIV infection was 25.50 ± 4.90 (range 18-42 years) and 25.37 ± 5.76 (range 16-45 years), respectively. Whereas mean age of asymptomatic HIV positive healthy non pregnant women and healthy HIV negative controls was 29.57 ± 6.77 (range 18-38 years) and 26.28 ± 5.9 (range 18-41 years), respectively, with no significant difference in age distributions (*P value *>0.05).

Table 1 shows the concentrations of serum retinol in pregnant women and non pregnant controls. After controlling for total serum protein, albumin and demographic variables, the mean ± SD serum vitamin A in HIV seropositive pregnant women (0.96 ± 0.42 μmol/L) was significantly lower than that in pregnant women without HIV infection (1.10 ± 0.45 μmol/L, P < 0.05). A significant interaction between HIV positive group and level of TSP was observed. To further explore this interaction, we compared the mean retinol concentrations of those with a high TSP and those with hypoproteinemia by HIV status. The mean retinol concentration was significantly lower in the HIV positive pregnant women with high TSP (P < 0.05) but was not significantly different from that of HIV negative group.

**Table 1:** Socio-demographic characteristics and serum parameters of pregnant and non pregnant women

Characteristics	Pregnant (n = 423)		Non pregnant (n = 55)	
	
	With HIV (44)	Without HIV (379)	With HIV (30)	Without HIV (25)
Age (years)	25.50 ± 4.90	25.37 ± 5.76	29.57 ± 6.7	26.28 ± 7.4
BMI (kg/m^2^)	23.56 ± 3.04^¶^	23.45 ± 3.05^¶^	18.67 ± 4.31	20.55 ± 3.1
Serum retinol (μmol/L)	0.96 ± 0.42*	1.10 ± 0.45^$^	0.74 ± 0.39^§^	1.18 ± 0.59
£Albumin gm/dl	3.65 ± 0.68*	3.99 ± 85	ND	ND
£Total protein (gm/dl)	6.12 ± 0.94	5.99 ± 1.22	ND	ND
£Cholesterol (mg/dl)	144.5 ± 54.20*	168 ± 51.52	ND	ND

**Keys**: ¶P value <0.001 versus HIV positive non pregnant women and HIV negative non pregnant women; *P value = 0.04 versus HIV negative pregnant women, ^$^P value = 0.02 versus HIV positive non pregnant women, ^§ ^P value = 0.000 verse HIV negative non pregnant women, ND: Not done, £ N = 107

The mean concentration of serum vitamin A was not significantly different between HIV positive pregnant women and HIV positive non pregnant women. It was also not significantly different between HIV negative pregnant women and HIV negative apparently health non pregnant controls. However, mean concentration of serum vitamin A level was significantly higher in HIV positive pregnant women (0.96+/-0.42) than that in HIV positive non pregnant women (0.74+/-0.39) (*P *= 0.02). Irrespective of pregnancy, HIV positive women had significantly lower serum concentration of vitamin A compared with HIV negative women (P < 0.05). Non pregnant women without HIV infection had significantly higher serum vitamin A concentration compared to non pregnant controls with HIV infection, P < 0.001. The mean serum vitamin A level of HIV positive pregnant women was 0.96 ± 0.42 where as the mean serum vitamin A level of HIV negative pregnant mothers was 1.1 ± 0.45. This mean difference in serum vitamin A level between the groups was statistically significant (*P = 0.01*).

Of the total 478 pregnant and non pregnant women (including controls), VAD was observed in 99 (20.7%) with a serum retinol level of less than 0.7 μmol/L (Table 2). The proportion of HIV positive pregnant women with serum retinol levels consistent with severe (0.00-0.34 μmol/L) and moderate (0.35-0.69 μmol/L) VAD was significantly higher than that in healthy controls (without HIV infection) and asymptomatic HIV infected controls (*p*< 0.05). From the total 74 HIV positive women, 33.8% (25/74) had a retinol level of less than 0.7 μmol/L and were vitamin A deficient.

**Table 2:** Vitamin A deficiency in pregnant and non pregnant women by HIV serostatus

Vitamin A Status	Pregnant women				Non pregnant women			
	
	HIV +ve	HIV -ve	OR (95%CI)	*P value*	HIV +ve	HIV -ve	OR (95%CI)	*P value *
VAD	11 (25)	67(17.7)	2.50(1.38-4.60)	0.002	14(46.7)	7(28)		
Non VAD	33 (75)	312(82.3)	1.30(1.14-1.50)	0.003	16(53.3)	18(72)		

**Keys: **+ve: Positive; -ve: Negative

Of 423 pregnant women, VAD was observed in 78 which accounted for 18.4% prevalence rate of VAD among pregnant women. Out of the 44 pregnant women with HIV infection, 11 (25%) were deficient for vitamin A with serum retinol level of below 0.7 μmol/L of which, 3 (6.8%) and 8 (18.2) had moderate (0.35-0.69 μmol/L) and severe (0.00-0.34 μmol/L) vitamin A deficiency, respectively. Similarly, among 30 asymptomatic HIV infected non pregnant voluntaries, 46.7% (14/30) were vitamin A deficient with serum retinol level of below 0.7 μmol/L, of which, 4 (13.3%) and 10 (33.3%) had moderate (0.35-0.69 μmol/L) and severe (0.00-0.34 μmol/L) vitamin A deficiency, respectively.

As indicated in Table 1 and 3, serum albumin and cholesterol concentrations were significantly lower in the HIV positive pregnant women compared with HIV negative pregnant women (P < 0.001). There was no significant difference in TSP value within the two groups (P = 0.06). Although the mean serum levels of vitamin A decreases with age, the difference between was not statistical significance (P = 0.07) (Table 4). Mean serum vitamin A level was significantly lower among women with BMI < 18.5 kg/m^2 ^than among those with BMI of 18.5 kg/m^2 ^or above, P value = 0.024 (Table 4). Although, BMI is not a surrogate for nutritional assessment during pregnancy, undernourished (BMI < 18.5 kg/m^2^) was observed in 4% of the pregnant women. Body weight and BMI were significantly lower in HIV infected pregnant woman compared to HIV non infected pregnant woman (P < 0.05). Undernourished (BMI < 18.5 kg/m^2^) was observed in 50% of asymptomatic HIV positive non pregnant woman (data not shown).

**Table 3:** Prevalence of micronutrient deficiencies and distribution of pregnant women and controls by HIV infection and serum status of micronutrients

Parameters	Cutoff value	HIV positive pregnant women (44)		HIV negative pregnant women (379)		HIV positive non pregnant women (30)		HIV negative non pregnant controls (25)	
		
		Deficient	Non deficient	Deficient	Non deficient	Deficient	Non deficient	Deficient	Non deficient
Vitamin A	0.7 μmol/L	33 (75)* 0.79 ± 0.32**	11(25) 1.45 ± 0.25	234 (61.7) 0.82 ± (0.26)^$^	145 (31.8) 1.54 ± (0.29)	26(86.7) 0.63 ± 0.28)	4(13.3) 1.45 ± (0.16)	12(48) 0.68 ± (0.27)	13(52) 1.6 ± (0.39)
TSP (107)	6.2 gm/dl	8/13(61) 5.6 ± (0.68)	5/13(39) 7.1 ± (0.35)	56/94(59) 5.2 ± (0.55)	38/94(41) 7.2 ± (0.85)	ND	ND	ND	ND
Albumin (107)	3.5 gm/dl	6/33 (18) 3.06 ± (0.39)	27/33(82) 3.04 ± (0.51)	7/74(9) 4.15 ± (0.39)	67/72(91) 4.3 ± (0.64)	ND	ND	ND	ND
Cholesterol(107)	154 mg/dl	9/13(69 119.8 ± (28)	4/13(31) 200.5 ± (59)	38/94(40) 119.5 ± (20.8)	56/94(60) 200.8 ± (38)	ND	ND	ND	ND

**Keys**: *Number (%), ** Mean ± SD, ^$^P value< 0.05 versus HIV negative non pregnant controls, ND: Not done

**Table 4:** Relationship between serum vitamin A level with age and body mass index (BMI)

Variables	N	Serum retinol (μmol/L) Mean ± SD	*P value*
Age (Years)			
< 20	20	1.09+0.44	0.070
21-30	274	1.06+0.45	
31-40	73	1.04+0.48	
41+	4	0.88+0.57	
BMI (kg/m^2^)			
<18.5	34	0.89 ± 0.43	0.024
>18.5	444	1.08 ± 0.45	

## Discussion

This cross-sectional study is the first of its kind in Ethiopian pregnant mothers with and without HIV infection in which serum levels of vitamin A has been measured. In this study, we found biochemical evidence that VAD was common in pregnant women, regardless of HIV status in Northwest Ethiopia. VAD has been defined as a public health problem when the prevalence of VAD, judged by serum retinol less than 0.7 μmol/l, among pregnant women is 15% [32]. In the present study, the overall prevalence of VAD among pregnant and non pregnant women was 18.4%, thus indicating a marginally major public health problem but neglected among these pregnant women in tropical settings of Northwest Ethiopia.

Due to multiple guidelines and lack of precise recommendation, nutritional surveys have used diverse reference values as a cut off indicating deficient vitamin A status. This in turn has jeopardized comparability of results of population-based prevalence studies. Although international consensus has yet to be establishes, serum retinol concentration below a cutoff of 1.05 μmol/l has been proposed to reflect low vitamin A status among pregnant and lactating women [21]. While the distribution of serum retinol concentrations below appropriate cut-offs are considered to reflect inadequate states of vitamin A nutriture, a low biochemical concentration of retinol in circulation is not considered a VAD. Thus, it is difficult to compare rates of VAD among previous studies. Even with this limitation, the 18.4% prevalence of VAD in this study population of pregnant women is a lower rate compared with previous report in a settings where about 40% rate of VAD has been reported [33] and previous reports from Bangladesh where VAD as high as 69% had been reported [34,35]. However, it is in agreement with a South African study which showed 16% VAD among mothers during the first 6 months of delivery [36]. The high rate of VAD in our study group could be due to dietary factors including inadequate vitamin A intake in Ethiopian population [24] and the relatively poor socio-economic and nutritional status of Ethiopian mothers compared with mothers in some other parts of developing nations.

This study showed that, HIV positive pregnant women had significantly lower mean serum retinol concentration than did HIV negative pregnant women (*P*<0.05). This finding was consistent with a study from Malawi [37], Rwanda [38,39] and Zimbabwe [40]. The low levels of serum vitamin A might be due to a low release of vitamin A from the liver during HIV infection, which would lead to low levels of vitamin A in the plasma despite the body having enough vitamin A liver stores [16]. The mean serum vitamin A level of HIV seropositive pregnant women was 0.96 ± 0.42 where as the level in HIV negative pregnant mother was 1.1 ± 0.45 (P value <0.001). This significant difference in serum vitamin A between HIV positive and negative pregnant women remained after measures of total serum protein, albumin and demographic variables (age, weight, BMI) were controlled for, which was also in agreement with a Zimbabwean study [40]. Low serum vitamin A level among HIV infected pregnant women was reported to be associated with a higher risk of vertical transmission of HIV [37-39], increased risk of preterm delivery and maternal anaemia [41].

HIV positive women had significantly lower serum concentration of vitamin A compared with HIV negative women *(P < 0.01*), irrespective of pregnancy. Serum retinol may transiently be low due to reduced production of retinol binding protein, which is often observed as part of the acute-phase response to infection [42]. Furthermore, it is also important to note that sub-clinical infection or inflammation has been found to be associated with the reduction of serum retinol level due to reduced production of retinol binding protein [42]. Although, we do not have any indication on the presence of sub-clinical infection on our study group and we did not measure acute inflammatory responses in the present study. It should be noted that, times of blood collections for all pregnant women was their routine antenatal follow up and all participants were healthy. Other possible confounding factors such as intestinal helminths [42,43] may influence serum retinol concentrations as high prevalence of helminthic infection was reported in the study area [26,27,43]. Low intake of green vegetables, fruit and dairy products could also be contributing factors for the low serum vitamin A level, as the staple food in Northwest Ethiopian is mainly a pancake named *enjera *made from a cereal called Tef (*Eragrostis teff*). Usually the *enjera *is eaten with stew made from legumes (mainly pea and beans).

In this study, overall prevalence of hypoalbuminaemia in pregnant women was relatively low (12%). This finding is similar to that of an earlier study carried out among Ethiopian pregnant mothers in the same locality [25]. No significant interaction between HIV sero-positive status during pregnancy and level of TSP was observed among vitamin A deficient women suggesting the lack of contribution of general protein-energy undernourished to VAD. In the present study, undernourished (BMI <18.5 kg/m^2^) women were found to have statistically significantly lower serum retinol level than well-nourished (BMI > 18.5 kg/m^2^) women. This is consistent with other studies from Bangladesh and Nepal [9,35].

## Conclusions

The present study shows that VAD among pregnant women in tropical settings of northwest Ethiopia is a major public health problem. Irrespective of pregnancy, HIV positive women had significantly lower serum concentration of vitamin A levels compared with HIV negative women. Considering the possible implications of VAD during pregnancy, we recommend multivitamin (which has a lower level of vitamin A) supplementation in the care and management of pregnant women with or without HIV infection.

### Limitations

This study has the following basic limitations. First, due to the presence of multiple guidelines and lack of precise recommendation, nutritional surveys have used diverse reference values as a cut off between normal and deficient vitamin A status. As a result, VAD in this study was defined based on WHO recommendation which are wildly accepted and used [1], as serum retinol level below 0.7 μmol/L. Second, micronutrient status is difficult to assess in the presence of infection, because biochemical indicators of several micronutrients are affected by the acute phase response. For instance, albumin, and retinol are "negative" responders, which increase during an acute phase response markers [36]. Thus, since in the present study, acute phase response (for example, C-reactive protein (CRP) and alpha 1- acid glycoprotein (AGP)) were not measured and controlled. Such markers were not measured in these subjects, due to the lack of laboratory facility. Hence, the results may be confounded by increased rates of acute phase response measures in the HIV positive women and may lead to an underestimation of vitamin A and albumin status. Third, body mass index (BMI) was used to determine the nutritional status of the study subjects though it is not a surrogate for nutritional assessment during pregnancy. Lastly, the low number of sample size among the control group resulted inflated HIV prevalence and hence might influence the results.

## Competing interests

No competing interests exist. This manuscript has not been published before or submitted elsewhere for publication.

## Authors' contributions

AM: conception of the research idea, study design, data collection and analysis and interpret the data and the draft of the manuscript; AK: conception of the research idea, study design, data collection and analysis, interpret the data and reviewed the manuscript; KH, BT, GY: data collection, part of laboratory work, data analysis and reviewed the manuscript; SY, MN, NN, FO: data analysis and interpretation and reviewed the manuscript; AB, YW: study design, interpret the data and reviewed the manuscript. All authors have read and approved the final version of the manuscript.

## Pre-publication history

The pre-publication history for this paper can be accessed here:

http://www.biomedcentral.com/1471-2458/11/569/prepub
